# The Relationship between Vitamin D, Inflammatory Markers, and Insulin Resistance in Children

**DOI:** 10.3390/nu16173005

**Published:** 2024-09-05

**Authors:** Omer Okuyan, Seyma Dumur, Neval Elgormus, Hafize Uzun

**Affiliations:** 1 Department of Pediatrics, Medicine Hospital, Faculty of Medicine, Istanbul Atlas University, 34408 Istanbul, Turkey; omer.okuyan@atlas.edu.tr; 2 Department of Medical Biochemistry, Faculty of Medicine, Istanbul Atlas University, 34408 Istanbul, Turkey; seyma.dumur@atlas.edu.tr; 3 Department of Microbiology, Faculty of Medicine, Istanbul Atlas University, 34408 Istanbul, Turkey; neval.elgormus@atlas.edu.tr

**Keywords:** children, vitamin D, insulin resistance, monocyte/HDL ratio, inflammatory index

## Abstract

Objective: In this study, we investigated 25-hydroxyvitamin D (25(OH)D, vitamin D), inflammatory hematologic ratios such as neutrophil-to-lymphocyte ratio (NLR), platelet-to-lymphocyte ratio (PLR), systemic immune-inflammation index (SII), monocyte/HDL-C ratio (MHR) and plasma atherogenic index (PAI) and possible relationships with insulin resistance (IR) in children. Methods: A total of 210 individuals, including 96 children with IR and 114 children without IR, aged 6–18 years, who were admitted to the Pediatric Endocrinology Outpatient Clinic at Medicine Hospital, Istanbul Atlas University were included in our study. Result: Compared to patients without IR, NLR, PLR, SII, and MHR were significantly higher in patients with IR. Fasting insulin, PAI, homeostasis model assessment of insulin resistance (HOMA-IR), and HOMA-β were significantly higher and quantitative insulin sensitivity check index (QUICKI) was considerably lower in patients with IR compared to those without IR. NLR, SII, and MHR were lower in normal vitamin D groups than the others (*p* < 0.001). PLR was lower in the group with normal vitamin D levels than the groups with insufficient or deficient levels of vitamin D (D < 21). Conclusions: We found that vitamin D deficiency in childhood is related to increased levels of circulating inflammatory markers (NLR, PLR, MHR, PAI), IR, and decreased insulin sensitivity. According to our results, supplementation of vitamin D may be beneficial in averting IR and enhanced systemic inflammation

## 1. Introduction

Childhood obesity has an increasing prevalence worldwide. Since adult obesity and obesity-related complications are more common in obese children and adolescents, childhood obesity has become an important health problem [1]. Dyslipidemia can be a consequence of obesity in both adults and children. Hyperinsulinemia increases triglyceride (TG) production from the liver. The most common dyslipidemia seen with obesity is increased TG levels and decreased high-density lipoprotein (HDL) levels. This is called atherogenic dyslipidemia [2,3].

Oxidative stress and inflammation are expressed by the proinflammatory effects of monocytes, the monocyte/HDL-C ratio (MHR), and are affected by the anti-inflammatory and antioxidant effects of HDL-C. This value has been used in many studies to determine whether inflammation and atherosclerosis contribute to the etiopathogenesis of cardiovascular diseases and type 2 diabetes mellitus (T2DM) [3,4,5,6]. Neutrophil/HDL-C ratio (NHR) is also an easily accessible potential index of inflammation [7].

25-hydroxyvitamin D (25(OH)D, vitamin D) deficiency has emerged as a widespread public health problem worldwide and is one of the most commonly undiagnosed nutrient deficiencies in all age groups [8]. Vitamin D levels are inversely associated with MHR among young medical staff [9]. Vitamin D plays a role in of insulin-sensitive tissues including the liver and skeletal muscle and can regulate insulin secretion. It may be associated with the pathogenesis of IR [10,11,12]. Since there are different conflicting studies [13,14,15] available, more studies need to be conducted to investigate the connection between vitamin D levels and IR in childhood.

Therefore, we investigated the mechanisms of IR, inflammation induction, and vitamin D deficiency associated with obesity in children. Understanding the impairment of diabetes-related insulin signaling induced by obesity may lead to better pharmacological strategies not only for the treatment but also for the prevention of obesity and diabetes. In this study, we investigated vitamin D, inflammatory hematologic ratios such as neutrophil-to-lymphocyte ratio (NLR), platelet-to-lymphocyte ratio (PLR), MHR, and plasma atherogenic index (PAI), and possible relationships with IR in children. 

## 2. Materials and Methods

### 2.1. Study Population

This cross-sectional study was conducted according to the guidelines of the Declaration of Helsinki and approved by the Istanbul Atlas University, Medical Faculty Clinical Research Ethics Committee (number of approval E-22686390-050.99-26195; Date: 13 April 2023). Data collection was completed prospectively. All patients and their parents signed an informed consent to execute the original measurements and to review their medical records. A total of 210 individuals, including 96 children with IR and 114 children without IR, aged 6–18 years, who were admitted to the Pediatric Endocrinology Outpatient Clinic at Medicine Hospital, Istanbul Atlas University were included in our study. All measurements were performed by healthcare professionals, who were blinded to the clinical diagnoses of the participants. 

### 2.2. Exclusion Criteria

Since we wanted to include only exogenously obese patients (without any other underlying disease) in the patient group, patients with hypothyroidism, Cushing’s disease, or syndrome that may cause obesity were excluded. In addition, patients with impaired glucose tolerance and DM were also excluded from the patient group without IR. Patients with communication problems, weakness, growth retardation, inflammatory diseases, infectious diseases, and those taking oral antidiabetic drugs, insulin, antihypertensive drugs and lipid-lowering drugs were excluded. Patients who were taking any nutritional supplements (vitamin D, iron, fish oil, prebiotics, probiotics, etc.), in the last three months were excluded.

### 2.3. Clinical and Biochemical Parameters

Height was measured with a Harpender stadiometer with a measurement accuracy of 0.1 cm and weight was measured with a SECA scale with a measurement accuracy of 0.1 kg. Participants’ weight was assessed after removing all clothing except underwear. 

The formula of weight (kg)/height^2^ (m^2^) was utilized to measure the body mass index (BMI) of the participants by dietitians using their height and body weight. 

Blood pressure was measured in a sitting position after at least 5 min of rest and as the average of three measurements.

For complete blood count and biochemical tests, blood samples were collected from the antecubital vein between 8 and 10 h in the morning at rest after 12 h of fasting. Blood samples taken for biochemical tests were centrifuged for 10 min and serum was obtained. To avoid possible assay variability, all patient blood samples were analyzed together.

Impaired fasting glucose was assessed by internationally defined criteria. Fasting plasma glucose ≤100 mg/dL was defined as normal, 100–125 mg/dL as impaired fasting glucose, and 75 g oral glucose tolerance test (OGTT), 2nd hour plasma glucose 140–199 mg/dL was considered as impaired glucose tolerance. OGTT was performed in patients with impaired fasting glucose and severe hyperinsulinemia [16].

For evaluation of IR, homeostasis model assessment of IR (HOMA-IR) index was utilized. HOMA-IR is calculated by the formula fasting insulin (uIU/mL) × fasting blood glucose (mg/dL)/405. Different cut-off values for IR were taken for prepubertal and pubertal periods (prepubertal HOMA-IR > 2.5 and pubertal HOMA-IR > 4) [17]. The status of IR in participants was also evaluated by using fasting glucose-to-insulin ratio (FGIR), quantitative insulin sensitivity check index (QUICKI), and HOMA-β [18,19]. 

Levels of 25(OH)D are interpreted as follows [20]: 21–29 ng/mL (52.5–72.5 nmol/L): vitamin D insufficiency; <20 ng/mL (<50 nmol/L): vitamin D deficiency.

Routine parameters such as glucose, cholesterol, and triglyceride were quantified with an automated analyzer (Cobas Integra 800; Roche Diagnostics GmbH: Mannheim, Germany). 

The levels of fasting insulin were determined using commercial kits and an automatic hormone analyzer (Beckman Coulter; Unicel DXI 600; Access Immunoassay System South Kraemer Boulevard Brea, CA, USA). The serum 25-hydroxyvitamin D [25(OH)D] levels were measured by enzyme-linked fluorescent assay on the Mini Vidas (Biomerieux, Paris, France). 

Serum high sensitive C-reactive protein (hs-CRP) was measured with chemiluminescent immunoassay using an ADVIA Centaur XP (Siemens Healthcare Diagnostics, New York, NY, USA).

Neutrophil/lymphocyte ratio (NLR) was calculated by dividing neutrophil count by lymphocyte count and monocyte/HDL ratio (MHR) was calculated by taking the ratio of monocytes to HDL. 

Plasma atherogenic index (PAI) was calculated from the logarithm of the ratio of triglyceride to HDL cholesterol.

### 2.4. Statistical Analyses

For data assessment and analysis, statistical Package for the Social Sciences version 21.0 software package for Windows (IBM Corp., Armonk, NY, USA) and Office 365 was used. Frequencies (n) and percentages (%) were used to show the descriptive characteristics of the data while numerical variables were described as mean ± standard deviation or median (25. percentile-75. percentile). A chi-square test was used to assess the distribution among categorical variables. To determine the distribution manner of the data, visuals (histograms and Q-Q plots), descriptive techniques (coefficient of variation, skewness, and kurtosis), and analytical methods (Kolmogorov–Smirnov Test) were employed. The independent samples *t* test or Mann–Whitney U test were utilized to compare continuous variables for two groups. To compare continuous variables between more than two groups *p* values one-way ANOVA or Kruskal–Wallis test were used. Adjusted *p* values and Tukey-HSD were utilized for post hoc significance. An ANCOVA analysis was performed using HOMA-IR or inflammatory markers as dependent variables and covariates, and vitamin D levels as the independent variable. To determine relationship between the numerical variables, the Pearson or Spearman correlation analyses were utilized. A *p*-value < 0.05 was considered as statistical significance.

## 3. Results

Of the participants, 54.3% (n: 114) did not have IR and 45.7% (n: 96) had IR. There was a statistically significant association relationship between IR and vitamin D deficiency. Among patients without IR, 61.4% (n: 70) had vitamin D in the normal range, 26.3% (n: 30) had vitamin D between 21 and 29, and 12.3% (n: 14) had vitamin D < 21. None of the patients with IR had normal vitamin D levels, 41.7% (n: 40) had vitamin D between 21 and 29, 58.3% (n: 56) <21 (Table 1). 

BMI and waist circumference were significantly higher, and vitamin D was significantly lower in the group with IR than without IR. BMI values in the sample ranged between 20.14 and 34.77, with a mean of 25.58 and a standard deviation of 3.22. The median BMI was 24.77, and the 25th and 75th percentiles were 23.37 and 27.45, respectively. Waist circumference ranged between 56 cm and 98 cm, mean of 74.16 cm, standard deviation of 9.37 cm, median of 75 cm, and 25th and 75th percentiles of 68 cm and 81 cm, respectively. Platelets and neutrophils were similar between IR groups. WBC, neutrophil, monocyte, CRP, total cholesterol, LDL cholesterol, VLDL cholesterol, triglyceride, and glucose levels were higher in the IR group than without IR; lymphocyte, lymphocyte percentage, and HDL cholesterol levels were lower (Table 2).

NLR, PLR, SII, and MHR were significantly higher in patients with IR compared to those without. Fasting insulin (19.25 (16.7–23.2) vs. 7.25 (5.49–9.3); *p* < 0.001), PAI (0.38 ± 0.12 vs. 0.23 ± 0.12; *p* < 0.001), HOMA-IR (4.56 (3.83–5.46) vs. 1.65 (1.26–2.08); *p* < 0.001), HOMA-β (233.09 (189.01–309.18) vs. 95.59 (74.63–125); *p* < 0.001) were significantly higher and QUICKI (0.31 ± 0.01 vs. 0.36 ± 0.02; *p* < 0.001) was considerably lower in patients with IR compared to those without IR (Table 2).

NLR, SII, and MHR were lower in normal vitamin D groups than the others (*p* < 0.001). PLR was lower in normal vitamin D than vitamin D < 21 groups. FGIR levels were significantly different between the three groups. FGIR was 14.83 (12.68–18.02) in the normal vitamin D group, 6.58 (5.12–8.84) in the vitamin D (21–29) group, and 5.19 (4.14–6.35) in the vitamin D < 21 groups. Fasting insulin and PAI levels were significantly different between the three groups. It was found that fasting insulin and PAI were lower in the normal vitamin D group compared to the others and higher in the vitamin D < 21 group compared to the others (Table 3).

QUICKI was significantly higher in the normal vitamin D group than the others (*p* < 0.001); HOMA-IR and HOMA-β were significantly lower (*p* < 0.001) (Table 3).

HOMA-IR as the dependent variable: For all inflammatory markers (NLR, PLR, SII, MHR, PAI), there are significant differences between vitamin D groups (*p* < 0.001). The partial eta squared values (0.301–0.474) indicate a moderate to large effect size, suggesting that vitamin D status explains a substantial portion of the variance in HOMA-IR. MHR and PAI are significant covariates (*p* < 0.001), while NLR, PLR, and SII are not (*p* > 0.05). Post hoc comparisons show significant differences between all vitamin D groups for most markers, except for PAI between groups 2 and 3.

Inflammatory markers as dependent variables: HOMA-IR is a significant covariate for MHR and PAI (*p* < 0.001), but not for NLR, PLR, and SII. There are significant differences between vitamin D groups for MHR and PAI (*p* < 0.001), but not for NLR, PLR, and SII. The effect sizes (partial eta squared) are smaller when inflammatory markers are the dependent variables (0.022–0.127) (Table 4).

PAI was strongly correlated with HOMA-IR (r: 0.682; *p* < 0.001), FGIR (r: −0.641; *p* < 0.001), QUICKI (r: −0.639; *p* < 0.001), moderately correlated with HOMA-β (r: 0.518, *p* < 0.001), MHR (r: 0.574; *p* < 0.001), weakly correlated with NLR (r: 0.256; *p* < 0.001) and SII (r: 0.269; *p* < 0.001), very weakly correlated with PLR (r: 0.174; *p*: 0.012) (Table 5).

HOMA-IR was very strongly correlated with FGIR (r: −0.952; *p* < 0.001), QUICKI (r: 1.0; *p* < 0.001), strongly correlated with HOMA-β (r: 0.781; *p* < 0.001) and MHR (r: 0.739; *p* < 0.001), moderately correlated with NLR (r: 0.447; *p* < 0.001) and SII (r: 0.434; *p* < 0.001), weakly correlated with PLR (r: 0.231; *p* < 0.001) (Table 5).

FGIR was very strongly correlated with HOMA-β (r: −0.926; *p* < 0.001), QUICKI (r: 0.952; *p* < 0.001), strongly correlated with MHR (r: −0.709; *p* < 0.001), moderately correlated with NLR (r: −0.438; *p* < 0.001) and SII (r: −0.421; *p* < 0.001), weakly correlated with PLR (r: −0.212; *p* < 0.001) (Table 5).

HOMA-β was strongly correlated with QUICKI (r: −0.781; *p* < 0.001), moderately correlated with MHR (r: 0.591; *p* < 0.001), weakly correlated with NLR (r: 0.358; *p* < 0.001) and SII (r: 0.333; *p* < 0.001), very weakly correlated with PLR (r: 0.139; *p* < 0.001).

MHR was weakly correlated with NLR (r: 0.351; *p* < 0.001), SII (r: 0.341; *p* < 0.001), and PLR (r: 0.222; *p* < 0.001) (Table 5).

## 4. Discussion

IR is a complex cellular disorder that affects multiple organ systems and leads to severe metabolic defects. In the current study, vitamin D was considerably lower in the IR group than in the group without IR. NLR, PLR, SII, and MHR were also higher in patients with IR compared to those without. NLR, SII, and MHR were lower in normal vitamin D groups than the others. PAI was strongly correlated with HOMA-IR. The importance of vitamin D levels should not be forgotten when monitoring and treating IR and related pathologies. Keeping vitamin D at optimal levels will be an inexpensive and easy preventive approach to metabolic control. Vitamin D functions as an immune modulator via monocytes and macrophages. Our findings suggest the supplementation of vitamin D may be helpful in metabolic control, and prevention of IR and related pathologies.

Vitamin D deficiency has recently become very common and has been associated with the pathogenesis of many diseases including metabolic abnormalities [21,22]. The relationship between vitamin D deficiency and IR is also gaining importance [23]. The results of our study showed that IR children had higher levels of vitamin D deficiency and insufficiency than non-IR children. Sharifi et al. [24] found that serum 25(OH)D levels are inversely associated with IR in children. Their results suggest that in metabolic syndrome (MetS) patients, HOMA-IR levels may be used as a cutoff value of 25(OH)D level in determining vitamin D deficiency. Moschonis et al. [25] showed that there was a negative correlation between serum 25 (OH) vitamin D levels and HOMA-IR levels and that children with IR had a higher prevalence of vitamin D deficiency and insufficiency compared to healthy age groups. Vitamin D receptors and metabolizing enzymes have been identified in most insulin-sensitive cell types like pancreatic cells and adipocytes [26]. However, the mechanism of vitamin D reducing the risk of developing metabolic disorders is not exactly discovered. Evidence suggests that vitamin D has a regulatory effect on pancreatic insulin secretion and blood glucose control. 

There are studies showing that Vit D has immunomodulatory and anti-inflammatory properties [27]. Vitamin D deficiency has been suggested to impair the immune system and cause infections. Markers of systemic inflammation are observed to increase in Vit D deficiency [28]. Vit D is known to have benefits in immune initiation, mucosal protection, and endothelial function. Vit D deficiency has also been associated with increased markers of systemic inflammation associated with multiple organ failure [29]. Reyman et al. [30] found a relationship between 25(OH)D deficiency, enhanced systemic inflammation, and reduced insulin sensitivity. In the current study, NLR, PLR, SII, and MHR were higher in patients with IR compared to those without. NLR, SII, and MHR were also lower in normal vitamin D groups than the vitamin D deficiency and insufficiency. HOMA-IR was strongly correlated with MHR, moderately correlated with NLR and SII, and weakly correlated with PLR. When we look at the biological basis of NLR increase, lymphocytes increase first in the immune response after hypertrophy of adipose tissues [31] and then lymphocytes produce cytokines such as TNF-alpha, IL-6, IL-1, IL-8, and adipokines (leptin, resistin, and visfatin) and mediate the recruitment of monocytes into adipose tissue and increase the number of neutrophils [32]. Thus, both neutrophils and lymphocytes increase in the early phase of inflammation with IR. In our study, CRP, an indicator of acute inflammation, increased in children with IR and vitamin D deficiency and insufficiency. In low-grade inflammation, monocytes are activated and some of them transform into lipid-loaded macrophages [33]. Thus, monocytes and macrophages trigger the formation or progression of cardiovascular diseases. In the study by Johnsen et al. [34], it was shown that increased monocyte levels were predictive of plaque development in arteries without prior plaque. In addition, there are many recent studies showing that HDL is effective in monocyte activation and inflammation in the development of atherosclerosis [35,36]. HDL has been found to be an anti-inflammatory molecule. Monocyte and HDL parameters may be indirect indicators of inflammation [37]. In our study, total cholesterol, LDL, VLDL, and TG levels were higher in the IR group than in the group without IR, and HDL was lower. MHR increased correlatively as HOMA-IR increased. Increased MHR is an expected result in proinflammatory backgrounds such as IR and vitamin D deficiency.

The PAI is a newly introduced index that reflects cardiovascular disease risk and dyslipidemia well [38]. In the current study, PAI was lower in the normal vitamin D group compared to the vitamin D insufficient or vitamin D deficient groups. Also, PAI was positively correlated with HOMA-IR, HOMA-β, MHR, NLR, and SII, while PAI was negatively correlated with FGIR and QUICKI. Vitamin D deficiency and IR are associated with waist circumference and BMI. This may be because large amounts of triglycerides and free cholesterol stored in body adipose tissue next to adipocytes are added to the circulation with increasing obesity. Thus, free circulating blood TG levels suppress hepatic lipoprotein lipase activity. As this enzyme is suppressed, circulating HDL begins to decrease. Low 25(OH)D levels are associated with increased IR and impaired lipid profile [39]. This may cause a tendency towards atherogenesis as well as an increase in PAI. The relationship between dyslipidemia and obesity may be explained by this mechanism [40]. The PAI value may contribute to the determination of cardiovascular risk in children with vitamin D deficiency, IR, and obesity by primary care physicians by being included in the laboratory result evaluation form without additional testing and cost.

It is possible to assess insulin sensitivity easily, quickly, and inexpensively in daily practice. Various tests have been described for this purpose and these methods have been found to show a strong correlation in the assessment of IR. Although the hyperinsulinemia euglycemic glucose clamp (HEGC) is considered the “gold standard” for determining peripheral IR, it is not used in routine clinical practice [41]. Several studies have found that it correlates with HOMA-IR [41,42,43]. Roth et al. [44] found that higher insulin, HOMA-IR, and HbA1c as well as lower QUICKI values were found in obese children with lower 25(OH)D concentrations even after adjustment for gender, age, and body mass index. Hypovitaminosis D is a risk factor for developing IR independent of adiposity. In a study conducted in Turkey [45], the HOMA-IR level and vitamin D deficiency were found to be secondary mediators in the development of dyslipidemia in obese children. In the current study, fasting insulin, HOMA-IR, and HOMA-β were significantly higher and QUICKI was significantly lower in patients with IR compared to those without IR. HOMA-IR was negatively correlated with FGIR, while HOMA-IR was positively correlated with QUICKI and HOMA-β. The HOMA-IR, QUICKI, HOMA-β, and FGIR tests are tests that can evaluate IR and insulin sensitivity in children in a practical way. Children with low 25(OH)D concentrations have lower insulin sensitivity (QUICKI). Even in those countries close to the equator, where sun exposure is generally assumed to be sufficient, serum vitamin D deficiency is a widespread health problem. Especially industrialized countries have implemented vitamin D supplementation to overcome vitamin D deficiency. A recent review has revealed the anti-inflammatory properties of vitamin D and its extra-skeletal activities. An important question has been the determination of 25(OH)D levels, which may influence IR and glucose metabolism and reduce the risk of developing disorders related to IR [46]. Identifying metabolic markers related to vitamin D and determining the treatment possibilities for these markers is one of the interesting topics that may be useful in the fight against IR in the future.

### Limitations of the Study

Factors affecting vitamin D levels such as climate, season, and lifestyle (dressing, sunbathing, diet) are not considered.

## 5. Conclusions

In vitamin D-deficient children, PAI value can be utilized as a marker to anticipate IR and inflammation. The PAI may be a potential therapeutic target in the treatment and prevention of IR in children. Vitamin D supplementation may impede the triggering effects of pro-diabetic, systemic inflammatory, and atherogenic pathways, which can be exhibited by high circulating inflammatory markers (NLR, PLR, SII, MHR) and PAI values. The HOMA-IR, QUICKI, HOMA-β, and FGIR tests can be used to practically evaluate IR and sensitivity in children. For the management of metabolic processes affected by IR, maintaining optimal vitamin D levels may be an acceptable practice that would support the diagnosis, follow-up, and treatment of these chronic diseases. Vitamin D deficiency in childhood is related to increased levels of circulating inflammatory mediators, IR, and decreased insulin sensitivity. Supplementation of vitamin D may be beneficial in averting IR and enhanced systemic inflammation, which is indicated by our results. Therefore, IR should be considered as a cluster of abnormalities that impair various physiological functions, rather than as a metabolic disorder alone.

## Figures and Tables

**Table 1:** The relationship between insulin resistance and vitamin D deficiency.

	Insulin Resistance Status				
	No Insulin Resistance (n: 114; 54.3%)		Insulin Resistance (n: 96; 45.7%)		
	n	%	n	%	*p* Value
Vitamin D (ng/mL)					
Normal vitamin D	70	61.4%	0	0%	
Vitamin D (21–29)	30	26.3%	40	41.7%	<0.001 *
Vitamin D < 21	14	12.3%	56	58.3%	
Vitamin D					
Normal vitamin D	70	61.4%	0	0%	<0.001 *
Vitamin D < 29	44	38.6%	96	100%	

*: Fisher’s exact test was applied.

**Table 2:** Relationship between insulin resistance and clinical and laboratory parameters and indexes.

	No Insulin Resistance	Insulin Resistance	
	Mean ± Std or Median (25p–75p)	Mean ± Std or Median (25p–75p)	*p* Value
Age (years)	11.91 ± 3.35	12.41 ± 3.29	0.139
Gender (M/F)	60/54	46/50	-
Body Mass Index (BMI) (kg/m^2^)	24.33 (23.14–26.02)	26.1 (23.71–28.28)	0.002 ^¥^
Waist circumference (cm)	69.9 ± 8.89	79.22 ± 7.17	<0.001 ^†^
Vitamin D (ng/mL)	31.1 (24–34.8)	18.7 (12.55–24)	<0.001 ^¥^
Systolic blood pressure (mmHg)	108 (103–115)	115 (108–127.5)	<0.001 ^¥^
Diastolic blood pressure (mmHg)	65 (63–70)	67 (63–71)	0.188 ^¥^
White blood cell (10^3^/µL)	7.54 (6.5–8.78)	8.34 (7.12–9.75)	0.009 ^¥^
Platelet (10^6^/µL)	304.01 ± 41.92	310.84 ± 40.07	0.231 ^†^
Lymphocytes (10^3^/µL)	2.91 ± 0.66	2.6 ± 0.76	0.002 ^†^
Lymphocytes (%)	36.99 ± 8.02	33.84 ± 10.23	0.013 ^†^
Neutrophil (10^3^/µL)	3.42 (2.71–4.41)	4.26 (3.36–5.3)	<0.001 ^¥^
Neutrophil (%)	52.1 (47.1–58.9)	54 (47.55–59.65)	0.182 ^¥^
Monocyte (10^3^/µL)	5.5 (4.9–6.4)	7.3 (5.85–8.3)	<0.001 ^¥^
Neutrophil/lymphocyte ratio (NLR)	1.18 (0.99–1.59)	1.6 (1.23–2.03)	<0.001 ^¥^
Platelet–lymphocyte ratio (PLR)	108.4 (87.83–120.78)	121.1 (96.5–150.85)	0.001 ^¥^
Systemic immune-inflammation index (SII)	366.61 (296.27–485.16)	510.76 (387.23–643.42)	<0.001 ^¥^
Monocyte/HDL cholesterol	11.66 (9.57–14.22)	18.62 (16.02–22.99)	<0.001 ^¥^
CRP (mg/L)	0.85 (0.5–1.54)	1.96 (0.91–2.77)	<0.001 ^¥^
Total cholesterol (mg/dL)	154.5 (147–165)	163 (149–180)	0.022 ^¥^
HDL cholesterol (mg/dL)	48.6 (42.4–51.2)	38.5 (33.4–43.9)	<0.001 ^¥^
LDL cholesterol (mg/dL)	89 (81–99)	106 (95–121.5)	<0.001 ^¥^
VLDL cholesterol (mg/dL)	17.2 (14–18.8)	18.5 (16.6–19.8)	<0.001 ^¥^
Triglyceride (mg/dL)	86 (70–94)	92.5 (83–99)	<0.001 ^¥^
Glucose (mg/dL)	90.68 ± 8.17	94.22 ± 9.97	0.005 ^†^
Glucose (mMol/L)	5.03 ± 0.45	5.23 ± 0.55	0.005 ^†^
Fasting insulin (µIU/mL)	7.25 (5.49–9.3)	19.25 (16.7–23.2)	<0.001 ^¥^
Plasma atherogenic index	0.23 ± 0.12	0.38 ± 0.12	<0.001 ^†^
HOMA-IR	1.65 (1.26–2.08)	4.56 (3.83–5.46)	<0.001 ^¥^
FGIR	12.68 (9.08–16.92)	5.08 (4.04–5.73)	<0.001 ^¥^
HOMA-B	95.59 (74.63–125)	233.09 (189.01–309.18)	<0.001 ^¥^
QUICKI	0.36 ± 0.02	0.31 ± 0.01	<0.001 ^†^

^†^: Independent samples *t* test; ^¥^: Mann-Whitney U test was applied.

**Table 3:** Relationship between vitamin D deficiency and clinical and laboratory parameters and indexes.

	Vitamin D (ng/mL)			
	Normal Vitamin D	Vitamin D (21–29)	Vitamin D (<21)	
	Mean ± Std or Median (25p-75p)	Mean ± Std or Median (25p-75p)	Mean ± Std or Median (25p-75p)	*p* Value
Age (years)	11.54 ± 3.41	12.31 ± 3.13	12.54 ± 3.29	0.066
Gender (M/F)	37/33	34/36	38/32	-
Body Mass Index (BMI) (kg/m^2^)	23.73 (22.64–24.46) ^a^	27.11 (25.62–28.25) ^b^	25.36 (22.66–29.04) ^c^	<0.001 ^¥^
Waist circumference (cm)	65.01 ± 6.21 ^a^	75.09 ± 5.29 ^b^	82.39 ± 6.71 ^c^	<0.001 ^†^
Systolic blood pressure (mmHg)	105 (102–108) ^a^	120 (110–125) ^b^	110 (108–130) ^b^	<0.001 ^¥^
Diastolic blood pressure (mmHg)	65 (63–70)	65 (60–75)	67 (65–70)	0.146 ^¥^
White blood cell (10^3^/µL)	7.49 (6.63–8.63) ^a^	8.02 (6.29–9.1) ^a,b^	8.39 (7.25–11.11) ^b^	0.016 ^¥^
Platelet (10^6^/µL)	309.3 ± 42.84	300.9 ± 52.26	311.2 ± 22.27	0.290 ^†^
Lymphocytes (10^3^/µL)	3.03 ± 0.56 ^a^	2.71 ± 0.67 ^b^	2.57 ± 0.84 ^b^	<0.001 ^†^
Lymphocytes (%)	38.62 ± 6.98 ^a^	34.54 ± 7.39 ^b^	33.46 ± 11.79 ^b^	0.002 ^†^
Neutrophil (10^3^/µL)	3.15 (2.53–3.71) ^a^	4.27 (3.06–5.2) ^b^	4.26 (3.44–5.27) ^b^	<0.001 ^¥^
Neutrophil (%)	51.2 (46–56.4)	53.95 (48.9–60.8)	53.6 (47.5–58.9)	0.158 ^¥^
Monocyte (10^3^/µL)	5.1 (4.6–6.1) ^a^	7.35 (6.3–8.5) ^b^	6.3 (5.3–7.5) ^c^	<0.001 ^¥^
Monocyte (%)	510 (460–610) ^a^	735 (630–850) ^b^	630 (530–750) ^c^	<0.001 ^¥^
Neutrophil/lymphocyte ratio (NLR)	1.06 (0.87–1.29) ^a^	1.59 (1.23–2.06) ^b^	1.57 (1.21–2.22) ^b^	<0.001 ^¥^
Platelet/lymphocyte ratio (PLR)	107.91 (86.3–117.86) ^a^	112.01 (90.78–131.73) ^a,b^	121.1 (102.85–145.24) ^b^	0.002 ^¥^
Systemic immune-inflammation index (SII)	328.44 (264–404.76) ^a^	486 (355.01–630.97) ^b^	498.72 (381.33–648.41) ^b^	<0.001 ^¥^
Monocyte/HDL cholesterol	10.41 (8.94–12.2) ^a^	18.4 (14.69–22.7) ^b^	17.06 (14.01–19.79) ^b^	<0.001 ^¥^
CRP (mg/L)	0.7 (0.4–1.1) ^a^	1.55 (0.95–2.35) ^b^	1.99 (0.88–2.89) ^b^	<0.001 ^¥^
Total cholesterol (mg/dL)	150 (145–157.5) ^a^	165 (152–181) ^b^	166.5 (149–186) ^b^	<0.001 ^¥^
HDL cholesterol (mg/dL)	49.8 (46.6–52.6) ^a^	41.1 (35.4–48.4) ^b^	39.5 (33.2–43.2) ^b^	<0.001 ^¥^
LDL cholesterol (mg/dL)	84.5 (78–90) ^a^	102.5 (95–121) ^b^	109.5 (97–124) ^b^	<0.001 ^¥^
VLDL cholesterol (mg/dL)	17 (14–18.4) ^a^	16.6 (14–19) ^a^	19 (17.8–19.8) ^b^	<0.001 ^¥^
Triglyceride (mg/dL)	85 (70–92) ^a^	83 (70–95) ^a^	95 (89–99) ^b^	<0.001 ^¥^
Glucose (mg/dL)	88.67 ± 6.4 ^a^	93.44 ± 9.66 ^b^	94.79 ± 10.03 ^b^	<0.001 ^†^
Glucose (mMol/L)	4.92 ± 0.36 ^a^	5.19 ± 0.54 ^b^	5.26 ± 0.56 ^b^	<0.001 ^†^
Fasting insulin (µIU/mL)	5.75 (5.05–7.3) ^a^	14.2 (9.7–19.8) ^b^	18.15 (15.3–22.2) ^c^	<0.001 ^¥^
Plasma atherogenic index	0.2 ± 0.11 ^a^	0.3 ± 0.14 ^b^	0.41 ± 0.1 ^c^	<0.001 ^†^
HOMA-IR	1.28 (1.13–1.63) ^a^	3.39 (2.13–4.83) ^b^	4.08 (3.54–5.23) ^b^	<0.001 ^¥^
FGIR	14.83 (12.68–18.02) ^a^	6.58 (5.12–8.84) ^b^	5.19 (4.14–6.35) ^c^	<0.001 ^¥^
HOMA-β (%)	84.63 (65.15–105.6) ^a^	170.44 (122.78–234.98) ^b^	206.44 (153.81–276.46) ^b^	<0.001 ^¥^
QUICKI	0.37 ± 0.01 ^a^	0.32 ± 0.02 ^b^	0.31 ± 0.02 ^b^	<0.001 ^†^

^†^: One-way ANOVA test; ^¥^: Kruskall–Wallis test were applied. a: <0.05; b: <0.01; c: <0.001.

**Table 4:** Relationship between vitamin D deficiency, HOMA-IR, and inflammatory markers.

Dependent Variable	Covariate	*p* Value	D Vit Group (*p* Value)	1 vs. 2	1 vs. 3	2 vs. 3	Partial Eta Squared
HOMA-IR	NLR	0.075	<0.001	<0.001	<0.001	0.001	0.433
HOMA-IR	PLR	0.228	<0.001	<0.001	<0.001	0.001	0.474
HOMA-IR	SII	0.064	<0.001	<0.001	<0.001	0.002	0.435
HOMA-IR	MHR	<0.001	<0.001	<0.001	<0.001	<0.001	0.304
HOMA-IR	PAI	<0.001	<0.001	<0.001	<0.001	0.239	0.301
NLR	HOMA-IR	0.075	0.009	0.091	0.006	0.320	0.045
PLR	HOMA-IR	0.228	0.103	1	0.196	0.179	0.022
SII	HOMA-IR	0.064	0.010	0.218	0.008	0.160	0.043
MHR	HOMA-IR	<0.001	<0.001	<0.001	1	<0.001	0.127
PAI	HOMA-IR	<0.001	<0.001	0.719	<0.001	<0.001	0.107

1: Normal Vitamin; 2: Vitamin D (21–29); 3: Vitamin D (<21).

**Table 5:** Relationship between indexes and insulin resistance indicators.

		HOMA-IR	FGIR	HOMA-B	QUICKI	Monocyte/HDL-C	NLR	PLR	SII
PAI	r	0.682	−0.641	0.518	−0.639	0.574	0.256	0.174	0.269
	p	<0.001	<0.001	<0.001	<0.001	<0.001	<0.001	0.012	<0.001
HOMA-IR	r		−0.952	0.781	−1.000	0.739	0.447	0.231	0.434
	p		<0.001	<0.001	<0.001	<0.001	<0.001	0.001	<0.001
FGIR	r			−0.926	0.952	−0.709	−0.438	−0.212	−0.421
	p			<0.001	<0.001	<0.001	<0.001	0.002	<0.001
HOMA-β:	r				−0.781	0.591	0.358	0.139	0.333
	p				<0.001	<0.001	<0.001	0.044	<0.001
QUICKI	r					−0.739	−0.447	−0.231	−0.434
	p					<0.001	<0.001	0.001	<0.001
Monocyte/HDL-C	r						0.351	0.222	0.341
	p						<0.001	0.001	<0.001
NLR	r							0.402	0.946
	p							<0.001	<0.001
PLR	r								0.529
	p								<0.001

Pearson correlation was used for the atherogenic index; QUCKI and Spearman correlation for the others.

## Data Availability

Participant-level data are available from the corresponding author.
